# Lurasidone induces developmental toxicity and behavioral impairments in zebrafish embryos

**DOI:** 10.3389/fpsyt.2025.1581524

**Published:** 2025-07-01

**Authors:** Wentian Li, Fang Wang, Zhe Feng, Qianqian Cheng, Yuqing Huang, Lisheng Zhu, Han Xiao, Hongjian Gong

**Affiliations:** ^1^ Department of Psychosomatic Medicine, Shanghai East Hospital, Tongji University School of Medicine, Shanghai, China; ^2^ Department of Psychosomatic Medicine, Wuhan Mental Health Center, Wuhan, Hubei, China; ^3^ School of Electrical Engineering and Automation, Hubei Normal University, Huangshi, China; ^4^ School of Mathematics and Physics, China University of Geosciences, Wuhan, China; ^5^ Institute of Maternal and Child Health, Wuhan Children’ s Hospital (Wuhan Maternal and Child Healthcare Hospital), Tongji Medical College, Huazhong University of Science and Technology, Wuhan, Hubei, China

**Keywords:** lurasidone, neurotoxicity, zebrafish embryo model, neurotransmitter systems, behavioral toxicology

## Abstract

**Introduction:**

Lurasidone, a second-generation antipsychotic widely used for schizophrenia and bipolar disorder due to its favorable metabolic profile, has poorly understood potential developmental neurotoxicity. This study investigated its effects on zebrafish embryos to address this gap.

**Methods:**

Zebrafish embryos were exposed to lurasidone at concentrations of 0, 0.4, 4, and 8 mg/L from 5 to 120 hours post-fertilization (hpf). We integrated morphological assessments, behavioral analyses, transcriptomic profiling, and neurotransmitter quantification to evaluate developmental neurotoxicity.

**Results:**

Lurasidone induced dose-dependent developmental toxicity, including reduced survival and hatching rates, decreased body length, and increased pericardial and yolk sac edema. Behavioral assessments revealed significant locomotor impairment and diminished touch response, especially at higher concentrations. Transcriptomic analysis identified 1,907 differentially expressed genes, with upregulation in circadian regulation pathways and downregulation in cell cycle and oxidative phosphorylation pathways. Neurotransmitter profiling showed significant reductions in glutamate, dopamine, and GABA levels.

**Discussion:**

These findings demonstrate lurasidone’s potential to disrupt neurodevelopment, suggesting perturbations in excitatory/inhibitory neurotransmitter balance and critical cellular pathways. The results highlight neurodevelopmental risks during sensitive periods, underscoring the need for further safety assessment in vulnerable populations (e.g., pregnant women, young patients).

## 1 Introduction

Lurasidone, a second-generation atypical antipsychotic, has emerged as a promising therapeutic option for both schizophrenia and bipolar disorder due to its unique receptor binding profile (1–3). The drug acts primarily through high-affinity antagonism at dopamine D2 and serotonin 5-HT2A receptors, while also showing affinity for other receptors including 5-HT7, 5-HT1A, and α2A-adrenergic receptors (4, 5). This complex receptor interaction profile contributes to its therapeutic efficacy in managing both positive and negative symptoms of schizophrenia, as well as bipolar depression. Notably, compared to other antipsychotics such as olanzapine and risperidone, lurasidone demonstrates minimal metabolic adverse effects, particularly regarding weight gain, glycemic abnormalities, and lipid elevations (6). This favorable metabolic profile has contributed to its increasing adoption in long-term psychiatric management, especially in metabolically vulnerable populations.

Despite its established clinical safety profile in adults, the potential developmental neurotoxicity of lurasidone, particularly during critical periods of neural development, remains inadequately investigated (7). Lurasidone increasingly prescribed for women of reproductive age due to its favorable metabolic profile (8), presents unique neuropharmacological characteristics including exceptional 5-HT7 receptor affinity (Ki=0.495 nM) that distinguishes it from other second-generation antipsychotics (9). Despite its FDA Pregnancy Category B designation, systematic evaluations of developmental neurotoxicity remain critically lacking, with documented pregnancy exposures being 18-fold fewer than olanzapine in major registries (10, 11). Compounding these concerns are its CYP3A4-dependent metabolism and high lipophilicity - pharmacokinetic properties that may potentiate fetal exposure through pregnancy-induced metabolic alterations (12) and enhanced placental transfer (13), creating urgent needs for dedicated developmental safety assessment. This knowledge gap is particularly concerning given that psychotropic medications can cross the blood-brain barrier and interact with key neurodevelopmental pathways. Previous studies have demonstrated that antipsychotic medications can potentially affect various aspects of neural development, including neurogenesis, synaptic plasticity, and neurotransmitter system maturation (14). Evidence from studies of other psychiatric medications suggests that even therapeutic doses may adversely affect neurodevelopment in animal models, leading to long-term behavioral and structural abnormalities. These findings underscore the importance of comprehensive evaluation of antipsychotic medications during critical developmental periods (15, 16).

The zebrafish (Danio rerio) has emerged as a powerful model for investigating developmental and neurobehavioral effects due to its unique advantages in developmental neurotoxicity studies (17–19). This model organism offers several key features: transparent embryos allowing real-time observation of developmental processes, rapid development with well-characterized developmental stages, and approximately 70% genetic homology with humans in disease-related genes (19). Particularly noteworthy is the zebrafish’s neural system development, which begins at 6 hours post-fertilization (hpf) and is largely complete by 6 days post-fertilization (dpf), enabling efficient observation of neurodevelopmental processes (20, 21). Previous studies have demonstrated zebrafish sensitivity to neuroactive compounds, with documented effects on embryonic development, locomotor behavior, and neurotransmitter systems (22, 23). For instance, research has shown that various psychiatric medications can affect zebrafish neurodevelopment through mechanisms involving oxidative stress, neurotransmitter disturbances, and altered gene expression patterns related to neural development (24, 25).

The assessment of developmental neurotoxicity is particularly crucial for psychiatric medications, as these compounds are designed to modulate neurotransmitter systems and can potentially interfere with normal neurodevelopmental processes (26). The developing nervous system exhibits unique susceptibilities to chemical perturbations, and effects during critical developmental windows may lead to permanent alterations in neural structure and function (27, 28). Understanding these potential impacts is essential not only for ensuring drug safety but also for optimizing treatment strategies in vulnerable populations, such as pregnant women and young patients (29). Recent advances in molecular and behavioral analysis techniques have enhanced our ability to detect subtle neurodevelopmental effects and elucidate their underlying mechanisms (30, 31).

In this study, we investigated the developmental toxicity and neurobehavioral effects of lurasidone using the zebrafish embryo model. Through a comprehensive approach combining morphological assessment, behavioral testing, transcriptomics, and neurotransmitter profiling, we aimed to (1) elucidate the potential impacts of lurasidone on early neurodevelopment, (2) identify molecular mechanisms underlying its developmental toxicity, and (3) provide critical insights into its safety profile during vulnerable developmental windows for improved risk assessment.

## 2 Materials and methods

### 2.1 Chemicals

Lurasidone (CAS: 150915-41-6, ≥98%) was purchased from TargetMol (T4576, United States). The experiment involved testing three specified concentrations of Lurasidone in fish water (0.4 mg/L, 4 mg/L, and 8 mg/L), with each concentration analyzed in three independent trials. The measured concentrations were verified via ultra-high-performance liquid chromatography paired with a triple quadrupole detector (UHPLC-MS/MS). Moreover, the stability of Lurasidone in fish water was examined by preparing three replicates of the four designated concentrations, which were stored under the exposure conditions (28°C with a 14-hour light/10-hour dark (14L:10D) cycle). Samples were collected at 0, 24, and 48 hours for concentration analysis.

### 2.2 Fish husbandry and embryo culture

Adult wild-type zebrafish were obtained from Exopet (Madrid, Spain) and housed in a regulated aquatic habitat maintained at 28 ± 1°C. Embryos were generated by natural spawning and subsequently collected using a sterile mesh strainer. These embryos were then cultivated in refreshed fish water with standardized physiochemical parameters: conductivity of 600 ± 55 µS/cm and pH 7.8 ± 0.2. They were incubated in a programmable thermostatic chamber set at 28.5°C, following a 14-hour light/10-hour dark (14L:10D) cycle, with a consistent density of one embryo per milliliter. According to the developmental staging criteria defined by (32), zebrafish embryos typically generally begin between 48 and 72 hours post-fertilization (hpf), transitioning to the larval stage at this point. Throughout the experimental period, embryos and larvae were maintained without additional feeding to prevent any potential influence on the Lurasidone exposure concentrations. All animal experiments were conducted in compliance with the Animal Ethics Code of Huazhong University of Science and Technology.

### 2.3 Lurasidone exposure and morphological abnormalities assessment

At 5 hpf, zebrafish embryos were systematically allocated into 6-well plates, with each well housing 30 embryos and 8 mL of either control or test solution. The study involved exposure to 0.01% dimethyl sulfoxide (DMSO) as a solvent control and three levels of lurasidone concentrations (0.4, 4, and 8 mg/L), kept at a steady temperature of 28 ± 0.5°C from 5 hpf to 120 hpf. Three biological replicates were established per group. Evaluations of mortality were c carried out at key developmental stages: 24, 48, 72, 96, and 120 hpf. Observations for malformations were conducted at 72 hpf, with deceased embryos removed daily. At 120 hpf, detailed assessments were performed, including the calculation of hatching rates.

### 2.4 Locomotive behavior analysis

The movement of zebrafish larvae at 120 hpf was thoroughly assessed using a Noldus behavior tracking system. The experimental setup featured a Pentax CCDIR XC E150 camera paired with a Pentax TV lens, mounted on a specially designed platform that included a lightbox with built-in LEDs and a light-blocking baffle to minimize external light interference. The light source provided both infrared (800–950 nm, peak at 860 nm) and visible (430–700 nm) illumination, with its spectral characteristics analyzed using a wideband spectroradiometer (RPS900, International Light Technologies, Peabody, MA). The intensity of the visible light was accurately measured at 69.5 lux via a photometer (model DR-2550-1, 2B silicon detector, TC284 photometric filter, Gamma Scientific, San Diego, CA).

The video signals from the camera were analyzed using a standard computer system equipped with Canopus Mediacruise MVR1000 software, producing MPEG-2 files for in-depth motion evaluation. Larvae were placed in 24-well plates, with one embryo in 4 mL of the test solution and six embryos per concentration and maintained at 28 ± 0.5°C. Following a 10-minute acclimation period, free-swimming behavior was documented recorded during two alternating 10-minute intervals of light and darkness.

At 5 dpf, zebrafish larvae underwent touch-response behavioral evaluations. For each test group, 12 larvae were randomly chosen for testing. Individual larva was positioned at the center of a circular plate (22 mm in diameter) filled with E3 medium. The touch stimulus was applied to the tail region of the larva with a fine needle. The subsequent swimming response was captured with a high-speed camera recording at 60 frames per second, following a modified protocol based on Basnet et al. (18). For quantitative assessment, the swimming area was segmented into four concentric zones: Zone 1 (5 mm diameter), Zone 2 (10 mm diameter), Zone 3 (15 mm diameter), and Zone 4 (20 mm diameter). The movement paths and zone distribution were analyzed to assess the escape response triggered by the touch. Each larva was tested only once to prevent habituation effects, and all experiments were performed at 28 ± 0.5°C.

### 2.5 Apoptosis detection

To assess the neurotoxicity of lurasidone on apoptosis in zebrafish embryos, acridine orange (AO) staining detection assays were conducted. At 120 hpf, embryos from each exposure group (n=12 per concentration) were washed thrice with phosphate-buffered saline (PBS) and stained in 1:5,000 AO solution for 30 minutes at 28°C under dark conditions. Following three additional PBS rinses (5 min each), embryos were anesthetized with 0.016% tricaine and mounted in 3% methylcellulose for imaging. Fluorescence visualization was conducted using an Olympus IX83 inverted microscope equipped with a 488 nm excitation filters. Quantitative analysis of apoptosis-associated fluorescence intensity was performed using ImageJ software (NIH, USA).

### 2.6 Transcriptomic profiling

Biochemical analyses indicated statistically significant disruptions in the medium-concentration (4 mg/L) lurasidone exposure group, leading to the selection of samples from both the control and medium-concentration groups for further transcriptomic studies. Upon completion of the 5-day experimental period, entire zebrafish larvae were meticulously collected, with each treatment group consisting of three separate biological replicates.

Total RNA was carefully isolated from brain tissue utilizing TRIzol reagent (Vazyme, Nanjing, China). The obtained high-quality RNA was then processed for library construction in preparation for next-generation sequencing. Raw sequencing data were performed on an Illumina NovaSeq 6000 sequencer, generating paired-end reads with a length of 2 × 150 base pairs. Functional analysis of differentially expressed genes (DEGs) was carried out through detailed Gene Ontology (GO) and Kyoto Encyclopedia of Genes and Genomes (KEGG) pathway enrichment analyses. All computational analyses were conducted using the Majorbio Cloud online bioinformatics platform (https://cloud.majorbio.com).

### 2.7 Detection of genes expression

Total RNA was extracted from zebrafish larvae at 120 hpf, utilizing a sample size of 30 replicated fish, with the TRNzol Universal Reagent Kit (Tinagen Biotech, Beijing, China). Complementary DNA (cDNA) was synthesized using the Hifair^®^ III 1st Strand cDNA Synthesis SuperMix for RT-qPCR Reagent Kit (Yeason Biotechnology, Shanghai, China), following the manufacturer’s instructions precisely. Quantitative real-time polymerase chain reaction (RT-qPCR) was conducted on a Quant Studio 3 platform (Thermo Fisher Scientific, Shanghai, China) using Hieff UNICON^®^ Universal Blue qPCR SYBR Green Master Mix (Yeason Biotechnology, Shanghai, China). The standardized thermal cycling protocol began with an initial denaturation phase at 95°C for 2 minutes, followed by 40 amplification cycles consisting of a 10-second denaturation step at 95°C and a 30-second annealing/extension step at 60°C. The final melting curve analysis involved sequential steps at 95°C for 15 seconds, 60°C for 1 minute, and 95°C for 1 second. Primer sequences used in the RT-qPCR analysis were detailed in **Supplementary Table S1**.

### 2.8 Targeted metabolomic analysis

Neurotransmitter-related metabolite analysis was performed utilizing liquid chromatography-electrospray ionization tandem mass spectrometry (LC-ESI-MS/MS) on a UHPLCQtrap system. The setup included an ExionLC AD system (SCIEX, USA) connected to an HSS T3 column (2.1 mm × 100 mm, 1.8 μm) and an AB SCIEX QTRAP 6500+ mass spectrometer (ABsciex, USA). Sample preparation followed the method outlined by Liu et al. (33).

The chromatographic separation was performed under carefully controlled conditions, with the column temperature set at 35°C and a consistent injection volume of 1 μL. The mobile phase comprised two components: mobile phase A (0.1% formic acid in water) and mobile phase B (0.1% formic acid in acetonitrile). Mass spectrometric settings were finely tuned to ensure accurate metabolite detection, including a spray voltage of +5500/−4500, a source temperature of 550°C, a curtain gas pressure of 35 psi, medium collision gas, ion source gas1 at 5, and ion source gas2 at 55. Data acquisition and processing were managed using integrated operating system packages. Comprehensive identification and quantification of the 17 metabolites were accomplished through the systematic use of authentic standards for each targeted metabolite.

### 2.9 Statistical analysis

Data analysis was carried out with the help of MATLAB R2021a software, with statistical significance assessed through Student’s t-test. Results are expressed as mean ± standard error (S.E.), with *P*-values less than 0.05 considered statistically significant. Each analysis was independently replicated a minimum of three times to ensure consistency and reliability.

## 3 Results

### 3.1 Lurasidone affected zebrafish early development

**Figure 1:**
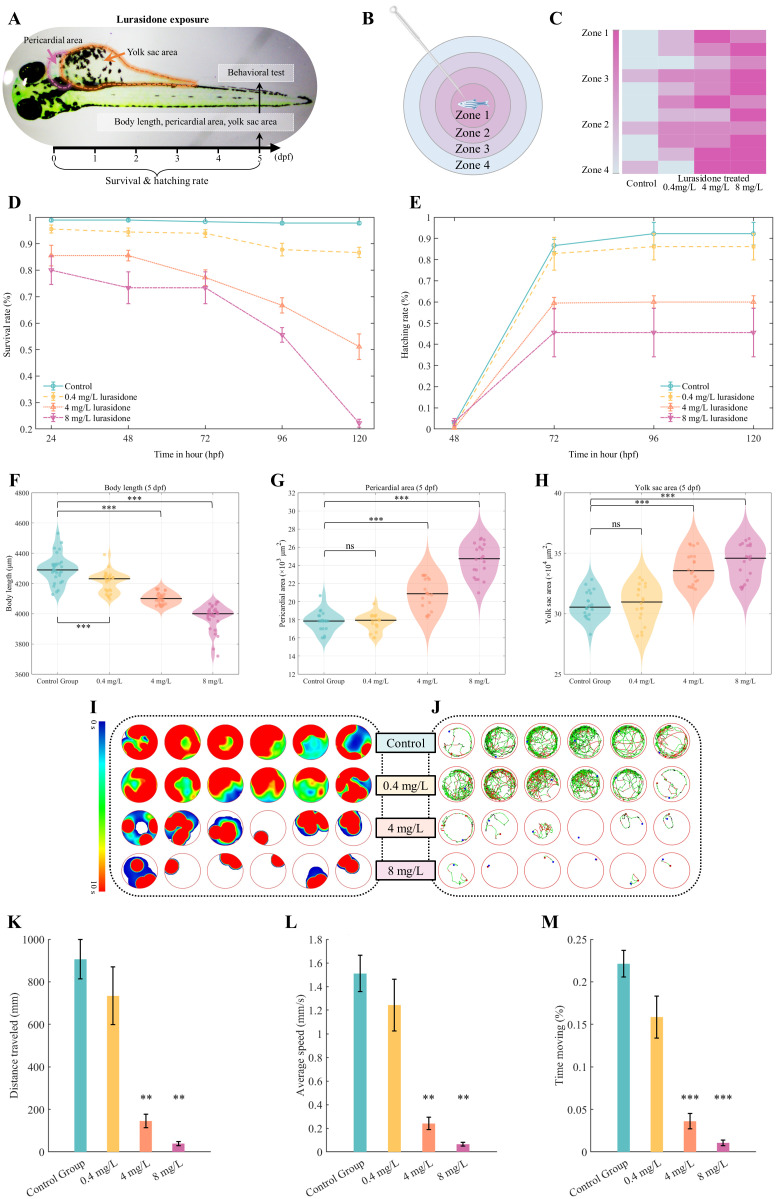
Developmental toxicity assessment of lurasidone exposure on zebrafish embryos. **(A)** Schematic illustration of the experimental design and morphological parameters measured in this study. **(B)** Schematic representation of zones and **(C)** heatmap visualization of touch-response patterns. **(D, E)** Temporal dynamics of survival and hatching rates from 1 to 5 days post-fertilization (dpf). **(F-H)** Morphometric analysis at 5 dpf showing body length, pericardial area, and yolk sac area measurements across groups. **(I)** Zone-specific residence time distribution and **(J)** representative locomotor trajectories following lurasidone exposure. **(K-M)** Quantitative behavioral parameters including total distance traveled, average swimming speed, and cumulative active movement duration. Data are presented as mean ± SE; statistical significance: *P < 0.05, **P < 0.01, ***P < 0.001 compared to control group; ns: not significant. No hatching was observed at 24 hours post-fertilization (hpf).

In this study, the developmental toxicity of the antipsychotic drug Lurasidone was systematically evaluated using a zebrafish embryo model. The experimental design was based on a dose-gradient scheme in which zebrafish embryos were exposed to 0 (control), 0.4, 4, and 8 mg/L of Lurasidone solution, and the developmental process was dynamically monitored by multidimensional indicators. As **Figure 1** shown, Lurasidone had a significant dose-dependent toxic effect on zebrafish embryonic development. The survival rate of the Lurasidone group exposed to 8 mg/L Lurasidone plummeted to about 20% after 120 hours, while the control group maintained a stable high survival rate (**Figure 1D**). Meanwhile, the hatching rate in the medium and high concentration Lurasidone-exposure groups (4 and 8 mg/L) also showed significant inhibition (**Figure 1E**), suggesting that the drug may have interfered with the normal developmental process of the embryos. Morphological analyses revealed significant developmental abnormalities caused by Lurasidone. The embryos showed a progressive decrease in body length with increasing Lurasidone concentration, while the pericardial region and yolk sac area increased abnormally. Such morphological changes were particularly evident in the medium and high concentration groups (4 and 8 mg/L), suggesting that Lurasidone may interfere with normal embryo growth by affecting organ development and nutrient metabolism.

### 3.2 Lurasidone affected motion of zebrafish larvae

In order to comprehensively assess the effects of Lurasidone on the motor behavior of zebrafish embryos, this study combined two dimensions of autonomous locomotion and touch response for systematic analysis (**Figures 1C, I-M**). The results of the touch-response experiment showed that the Lurasidone-treated groups showed a significant change in the pattern of response to mechanical stimuli. Control group showed rapid and directed avoidance responses to touch stimuli, with activity trajectories concentrated in Zone 1 and Zone 2 regions. The medium concentration group (4 mg/L) showed a significant delay in response, and avoidance behavior was significantly affected in terms of both direction and distance. The high concentration group (8 mg/L) almost lost the ability to respond to touch stimuli and had difficulty in inducing effective avoidance behavior even when given stimuli.

In terms of voluntary locomotion, the experimental data also showed a clear dose-dependent inhibitory effect. The control embryos showed normal locomotor ability, with an average swimming distance of about 906 mm, an average swimming speed maintained at about 1.51 mm/s, and a locomotion time ratio of 0.22. Motor parameters showed a significant decrease with increasing concentrations of Lurasidone. All three indicators of the medium concentration group were significantly reduced (p < 0.01), swimming distance decreased to about 145mm. The zebrafish embryos in the high concentration group almost completely lost motor function, with a swimming distance of less than 50 mm. This integrated behavioral change model suggested that Lurasidone not only affected autonomic locomotion but also interfered with the functioning of the sensory-motor integration system and Lurasidone might act on both sensory and motor neuron development.

### 3.3 Lurasidone induces concentration-dependent apoptosis

**Figure 2:**
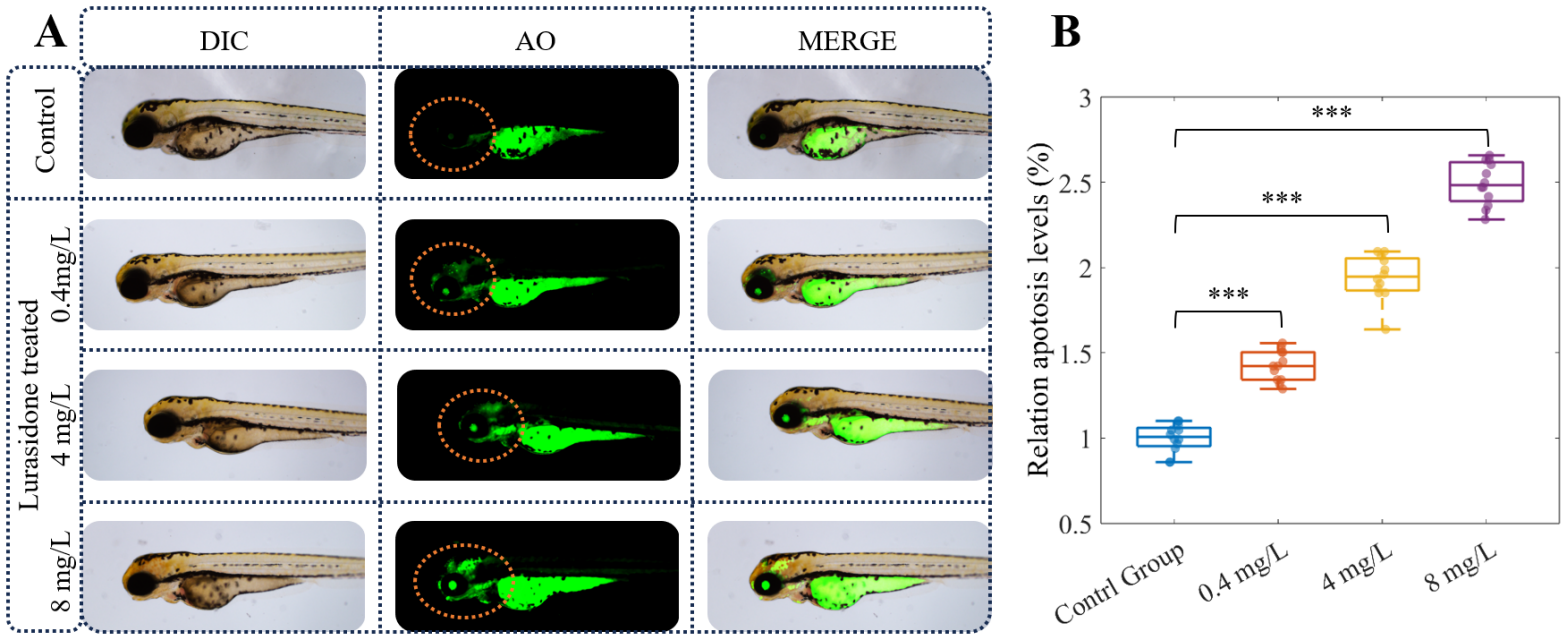
Lurasidone induces dose-dependent neuronal apoptosis in zebrafish embryos. **(A)** Representative images of 120 hpf zebrafish embryos under differential interference contrast (DIC) and fluorescence microscopy following acridine orange (AO) staining. Embryos were exposed to different concentrations of Lurasidone (0.4, 4, and 8 mg/L) or vehicle control. Apoptosis was visualized by acridine orange staining in live embryos, where apoptotic cells are indicated by green fluorescence, with merged images displaying the anatomical localization of apoptotic signals. **(B)** Quantitative analysis of relative apoptosis levels (%) across treatment groups. Data are presented as box plots showing median, quartiles, and individual data points (n=12 per group). Statistical significance is indicated by asterisks (***: *P* < 0.001) compared to control group.

The AO staining assay revealed a dose-dependent increase in neuronal apoptosis in zebrafish embryos exposed to lurasidone (**Figure 2**). Fluorescence microscopy imaging demonstrated progressively enhanced AO-positive signals in the central nervous system of 120 hpf embryos following lurasidone treatment at concentrations of 0.4, 4, and 8 mg/L compared to the control group. Quantitative analysis of fluorescence intensity confirmed significant elevation in apoptotic levels across all treatment groups, with relative apoptosis levels of 1.4228 ± 0.1, 1.9418 ± 0.3, and 2.4922 ± 0.2 for the 0.4, 4, and 8 mg/L exposure groups, respectively, compared to controls normalized to 1.0 (*p* < 0.001). Notably, the highest concentration (8 mg/L) induced approximately 2.5-fold increase in neuronal apoptosis relative to untreated embryos. The apoptotic signals were predominantly localized in the midbrain and hindbrain regions, with minimal effect observed in peripheral tissues, suggesting neuronal specificity of lurasidone-induced cytotoxicity. These findings indicate that lurasidone exposure elicits concentration-dependent neurotoxic effects in developing zebrafish, potentially through mechanisms involving programmed cell death pathways in neural tissues.

### 3.4 Lurasidone induced transcriptomic alterations in zebrafish larvae

#### 3.4.1 Overview of differentially expressed genes

**Figure 3:**
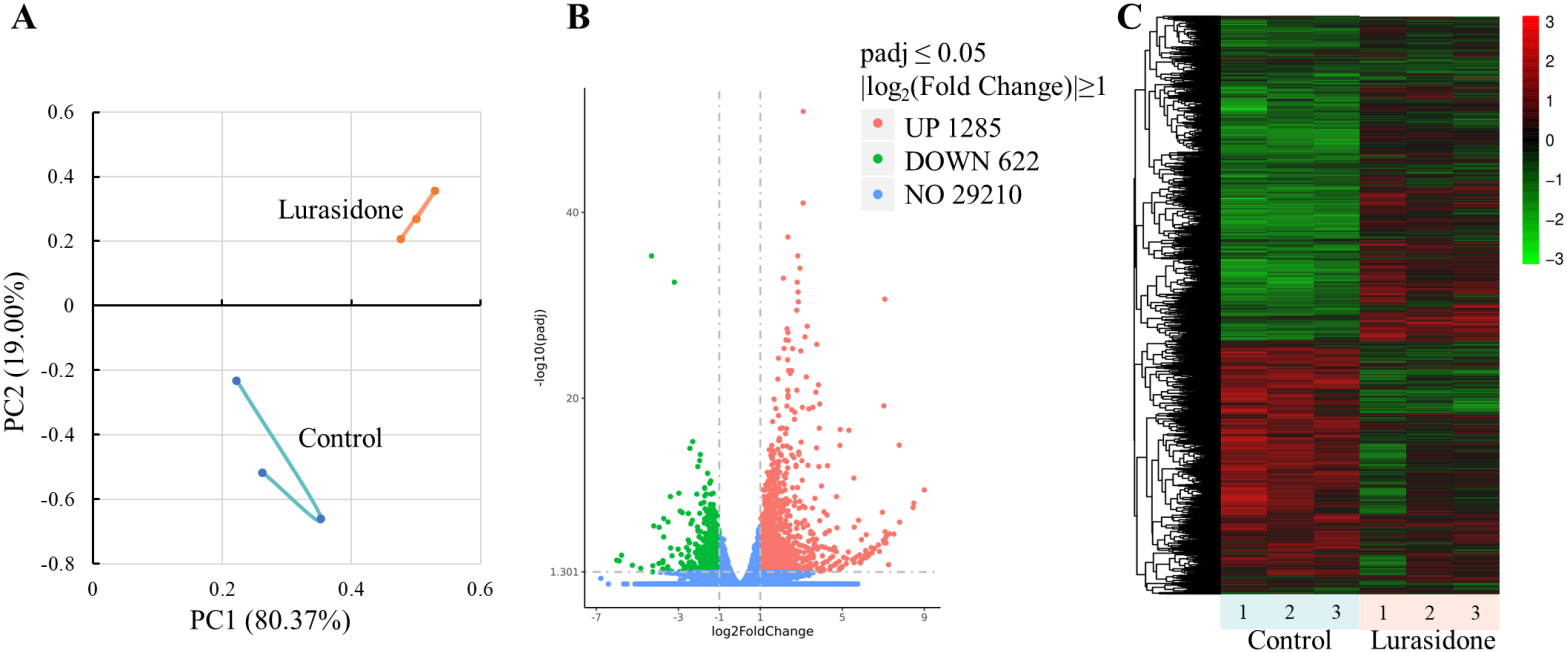
Transcriptome analysis of zebrafish embryos exposed to Lurasidone. **(A)** Principal component analysis (PCA) showed distinct clustering of control and Lurasidone-treated (4 mg/L) groups based on global gene expression profiles. **(B)** Volcano plot displaying differentially expressed genes (DEGs). The threshold criteria were set at padj ≤ 0.05 and |log2(Fold Change)| ≥ 1. **(C)** Hierarchical clustering heatmap of DEGs across all samples.

In this study, the effects of Lurasidone (4 mg/L) on zebrafish embryonic development were investigated by transcriptome sequencing analysis. The results of principal component analysis (PCA) showed that the control and Lurasidone-treated samples showed a significant trend of separation in the PC1 and PC2 dimensions (**Figure 3A**). The first principal component (PC1) explained 80.37% of the variance and the second principal component (PC2) explained 19.00% of the variance, suggesting that Lurasidone treatment resulted in significant transcriptome level alterations. This high level of sample differentiation suggested a systematic effect of Lurasidone on zebrafish embryonic gene expression patterns. The results of the differentially expressed gene (DEG) analysis (**Figure 3B**) showed that a total of 1,907 DEGs were identified. Among them, 1,285 genes were up-regulated and 622 genes were down-regulated, while 29,210 genes showed no significant change in expression. This result indicated that Lurasidone treatment induced the up-regulation of more genes.

#### 3.4.2 GO term and KEGG pathway analysis

Functional enrichment analysis based on transcriptomic data showed that Lurasidone treatment had a significant effect on the regulation of gene expression in zebrafish embryos (**Figure 3**). GO enrichment analysis showed that significantly up-regulated genes after Lurasidone treatment were mainly involved in circadian regulation, while significantly down-regulated genes were focused on cell cycle processes (**Figures 3A, B**). In terms of biological processes (BP), up-regulated genes were mainly enriched in circadian-related biological processes, including regulation of circadian rhythm (GO:0042752), circadian rhythm (GO:0007623) and rhythmic process (GO:0048511). Particularly, they were significant in the circadian regulation of gene expression (GO:0032922) and entrainment of the circadian clock by photoperiod (GO:0043153). In addition, these genes are involved in biological processes such as muscle contraction (GO:0006936) and negative regulation of endopeptidase activity (GO:0010951). Down-regulated genes were significantly enriched for cell cycle-related functions, including cell cycle process (GO:0022402) and mitotic cell cycle (GO:0000278). In terms of cellular components (CC), up-regulated genes were significantly enriched in cellular components associated with muscle structure. The most significant were myosin filament (GO:0032982) and myosin II complex (GO:0016460). Down-regulated genes were significantly enriched in CC related to mitochondrial structure, such as the inner mitochondrial membrane protein complex (GO:0098800) and mitochondrial membrane part (GO:0044455). In terms of molecular function (MF), up-regulated genes were mainly enriched in the functions of tetrapyrrole binding (GO:0046906), microfilament motility activity (GO:0000146), and heme binding (GO:0020037). These results suggested that up-regulated genes were mainly involved in biological processes related to circadian regulation and muscle tissue function, and played important roles in cellular structural organization and metabolic enzyme activity. And Lurasidone may interfere with essential processes of embryonic development by inhibiting cell cycle and mitochondria-related functions.

In the KEGG pathway enrichment analysis (**Figure 3C**), up-regulated genes were significantly enriched in several signaling and metabolic pathways. Among them, the Adipocytokine signaling pathway (dre04920) and FoxO signaling pathway (dre04068) were significantly enriched. These pathways are closely related to embryonic metabolic regulation, oxidative stress response and cell survival. The down-regulated genes were significantly enriched in the Cell cycle (dre04110), Oxidative phosphorylation (dre00190) and Ribosome pathways (dre03010). The inhibition of these pathways suggests that Lurasidone may significantly affect embryonic growth and development by blocking embryonic cell proliferation and protein synthesis and inhibiting mitochondrial energy metabolism.

The effects of Lurasidone on zebrafish embryos showed multi-level and multi-directional regulation. The up-regulated genes mainly affected metabolism, circadian rhythms and muscle tissue-related biological processes, suggesting that Lurasidone may play a role in metabolic regulation and rhythmic development of the embryo; whereas the down-regulated genes were concentrated in the pathways of basic life activities, such as the cell cycle, oxidative phosphorylation, and protein synthesis, suggesting that Lurasidone may interfere with normal processes of embryo development by inhibiting energy metabolism and cell proliferation. interfere with the normal process of embryonic development.

#### 3.4.3 The verified results of RNA-Seq by RT-qPCR

**Figure 4:**
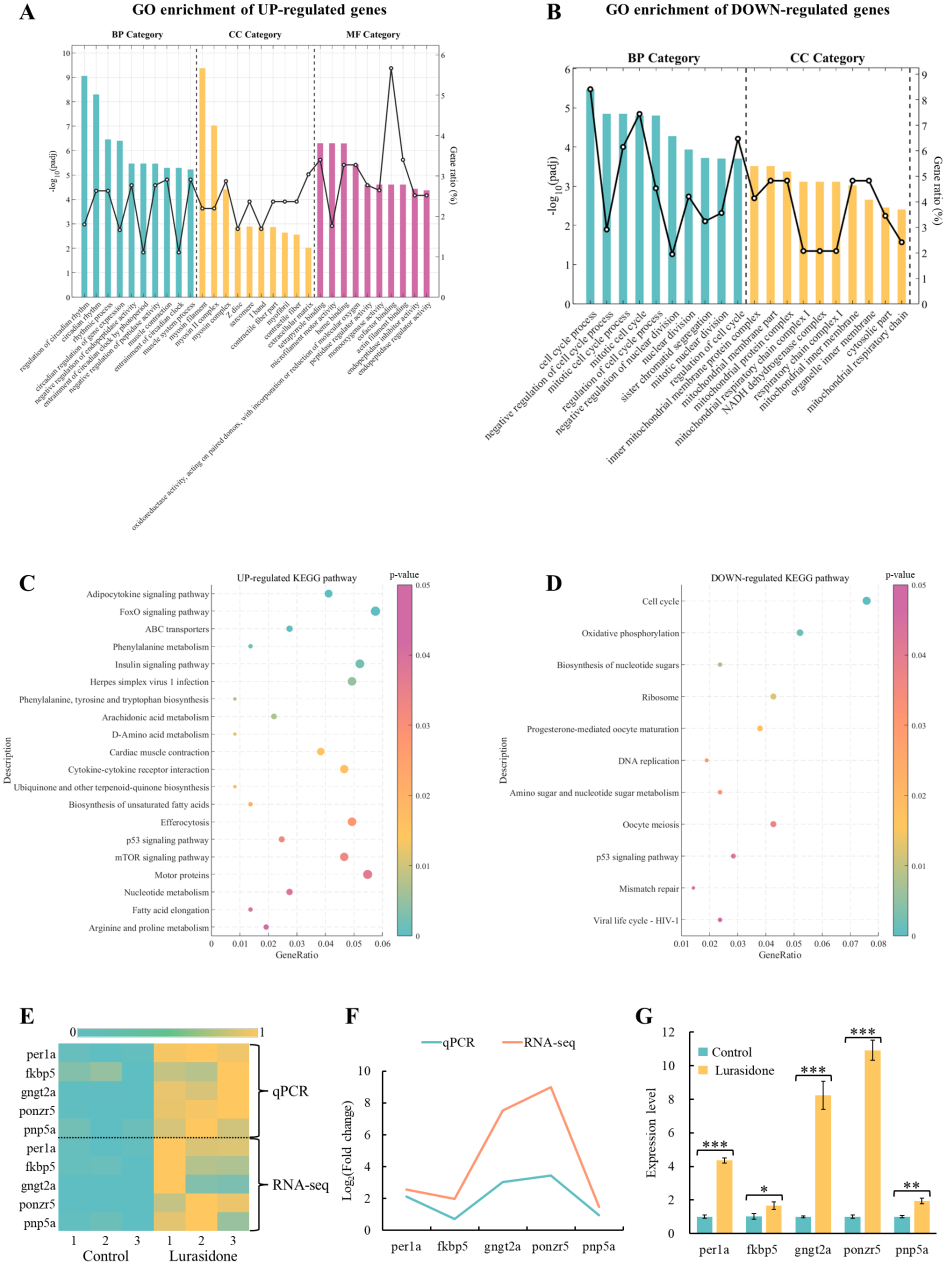
GO enrichment and KEGG pathway analysis of differentially expressed genes (DEGs), and transcriptional and qPCR validation of key genes in Lurasidone-treated zebrafish embryos. **(A, B)** Top 10 GO terms for up-regulated and down-regulated DEGs categorized into biological processes (BP), molecular functions (MF), and cellular components (CC). Adjusted p-values (padj) were used to assess statistical significance. **(C, D)** KEGG pathway enrichment analysis of up-regulated and down-regulated DEGs with p-values indicating pathway significance. **(E)** Heatmap of transcriptomics data displaying normalized expression levels of selected key genes. **(F, G)** qPCR validation results for the same set of genes, presented as mean ± S.E. with statistical significance denoted as *P < 0.05, **P < 0.01, and ***P < 0.001.

Based on the results of GO and KEGG enrichment analyses above, the genes per1a, fkbp5, gngt2a, ponzr5 and pnp5a were selected for validation in this study (**Figures 4E–G**). In the GO enrichment analysis of up-regulated genes, circadian rhythm and rhythmic process were significantly enriched BP, suggesting that the circadian pathway is activated after Lurasidone treatment. per1a is a core gene in the circadian rhythm pathway (CRP), which is involved in the maintenance and regulation of circadian rhythms. The significant up-regulation of per1a supported the activation of circadian rhythm-related pathways (circadian rhythm) in the transcriptomic data. Among the KEGG-enriched up-regulated genes, there was a significant enrichment of the FoxO signaling pathway and the Adipocytokine signaling pathway, which are closely related to stress response and metabolic regulation in embryos. fkbp5 is a key gene in the stress response and hormone signaling pathway that regulates hypothalamic-pituitary-adrenal axis (HPA axis) activity. Up-regulation of fkbp5 was verified to support Lurasidone-induced stress response in embryos, validating the potential disruption of the HPA axis by Lurasidone. gngt2a is a component of the G protein signaling pathway involved in cell signaling, retinal function and metabolic regulation. Validation of the expression of gngt2a could further supported the effect of Lurasidone on the regulation of embryonic signaling pathways, in particular G-protein-coupled receptor-mediated metabolic and hormonal regulation. ponzr5 functions during cell division and structural reorganization and may be relevant to the regulation of embryonic development. Unlike the down-regulation of cell cycle-related genes such as ccnb1 and cdk1, the up-regulation of ponzr5 may suggest a different mode of regulation of metabolic and embryonic structural regulatory genes. pnp5a is a key gene in purine metabolism, which is mainly involved in purine nucleotide metabolism, and its role is very important for nucleic acid synthesis, energy metabolism and cell proliferation. According to KEGG enrichment analysis, Nucleotide metabolism and Biosynthesis of nucleotide sugars were significantly enriched pathways, suggesting that the upregulation of pnp5a is closely related to these metabolic pathways. pnp5a, like ponzr5, may play a positive role in the regulation of embryonic structure and metabolism.

The mechanism of action of lurasidone is complex and multilayered, and may affect embryo development in an integrated manner through a dynamic balance between metabolism, structural regulation, and the cell cycle.

### 3.5 Lurasidone induced neurotransmitter alterations in zebrafish larvae

#### 3.5.1 Overview of differentially expressed neurotransmitters

**Figure 5:**
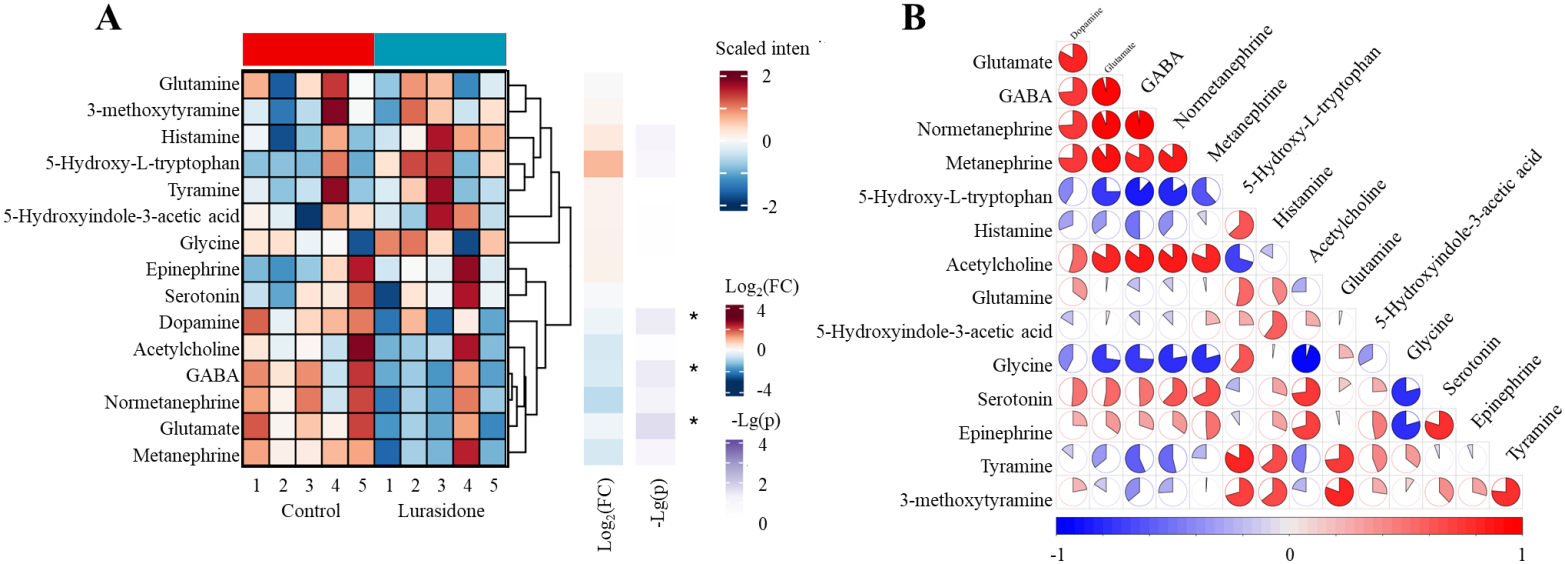
Systematic alterations of 15 neurotransmitters in zebrafish larvae following lurasidone exposure. **(A)** Hierarchical clustering heatmap showing differential expression patterns of neurotransmitters. **(B)** Correlation network analysis of neurotransmitter changes. Significance levels: *p* < 0.05 denoted by *; not marked * indicates not significant]. Data are presented as mean ± SE. (FC: Fold change).

The effect of Lurasidone on zebrafish embryos neurotransmitter metabolism caused several significant changes. As **Figure 5** showed, among the major neurotransmitters, statistically significant decreases were observed in the levels of glutamate, dopamine and GABA, with decreases of 11%, 11% and 29%, respectively. This pattern of change was shown as a distinct blue area in the heatmap, reflecting the significant down-regulation trend of these substances. From a metabolic pathway perspective, the glutamate-GABA system was significantly affected, with both key neurotransmitters showing significant down-regulation, suggesting that Lurasidone may have affected the excitatory/inhibitory neurotransmitter balance. Although these changes align with lurasidone’s established pharmacological profile as an antagonist of dopamine D2, 5-HT2A, and 5-HT7 receptors, the disproportionate reduction in GABA levels (29%) compared to glutamate and dopamine (both 11%) represents a novel finding that extends beyond what would be predicted from receptor binding profiles alone. This substantial GABA reduction suggests previously uncharacterized downstream effects on inhibitory neurotransmission pathways.

The observed neurotransmitter changes provide a mechanistic link between transcriptome alterations and behavioral deficits. Significant reductions in glutamate and GABA levels may explain the impaired tactile response and reduced motor ability, as these neurotransmitters are essential for sensorimotor integration and motor control. Simultaneous alterations in inhibitory (GABA) and excitatory (glutamate) neurotransmission suggested that lurasidone affected the fundamental balance of neural signaling. This observation was consistent with the enrichment of circadian regulation and muscle contraction-related pathways shown by transcriptomic data, suggesting that lurasidone comprehensively affected neural signaling and motor function through multiple mechanisms. While these findings align with lurasidone’s known pharmacological profile as an antagonist of dopamine D2 receptors, our results reveal for the first time its significant impact on the glutamate-GABA system *in vivo*, particularly the substantial reduction in GABA (29%), which extends beyond what would be expected from its established receptor binding profile alone. This pronounced effect on inhibitory neurotransmission represents a novel aspect of lurasidone’s mechanism not previously characterized in developmental models.

#### 3.5.2 Analysis of absolute quantitative results of neurotransmitters

**Figure 6:**
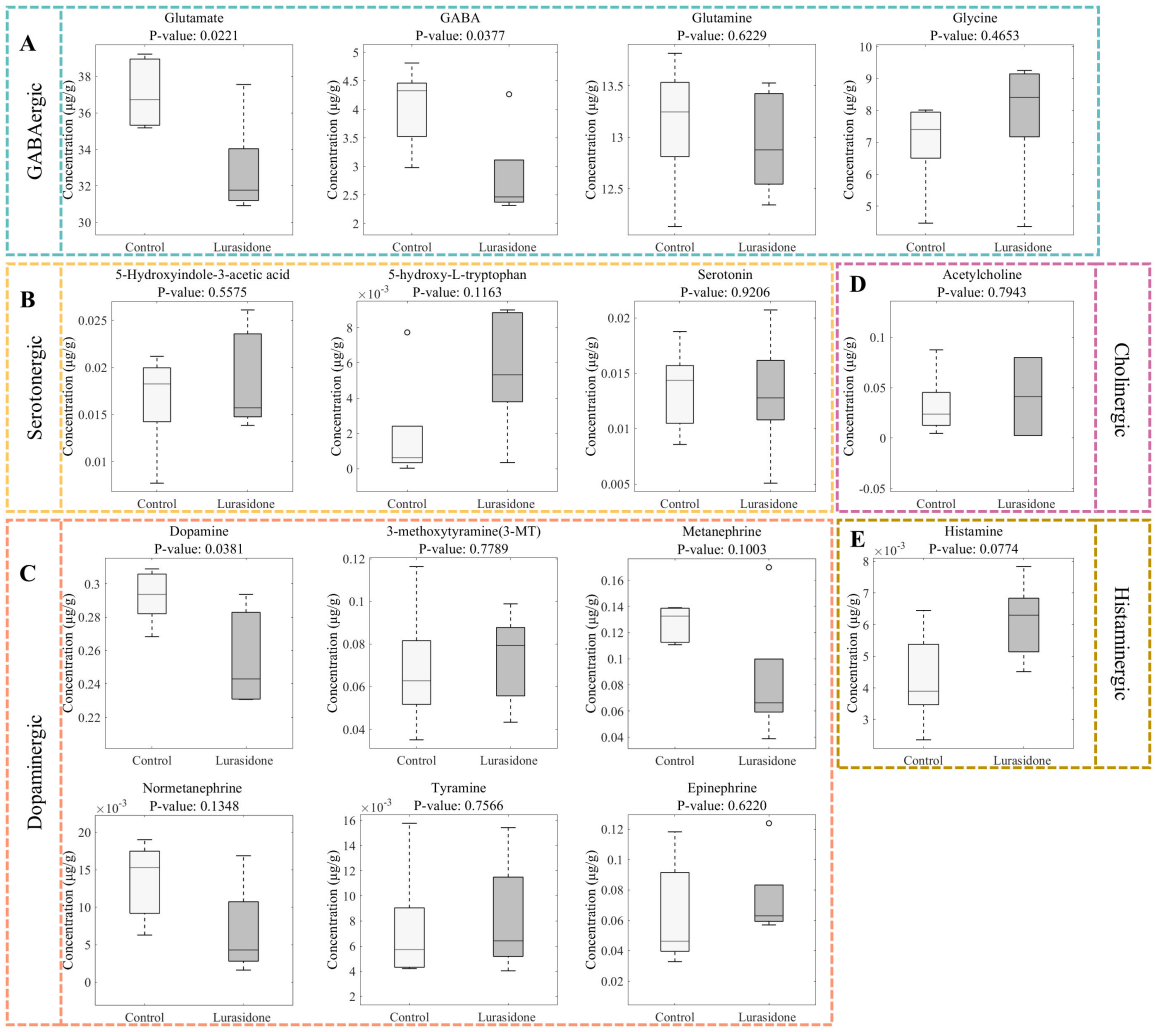
Quantitative analysis of neurotransmitter alterations in zebrafish larvae following lurasidone exposure. **(A)** Box plots showing changes in GABAergic system components (glutamate, GABA, glutamine, and glycine). **(B)** Alterations in serotonergic pathway metabolites (5-hydroxyindole-3-acetic acid, 5-hydroxy-L-tryptophan, and serotonin). **(C)** Changes in dopaminergic system components (dopamine, 3-methoxytyramine, metanephrine, normetanephrine, tyramine, and epinephrine). **(D)** Effect on cholinergic neurotransmitter (acetylcholine). **(E)** Impact on histaminergic system (histamine). *P*-values were calculated using Student’s t-test. Data are presented as mean ± SE.

**Figure 6** shows the results of quantitative analysis of neurotransmitter changes in zebrafish larvae after exposure to Lurasidone. In the glutamatergic system, glutamate concentration was significantly reduced from 37.07 ± 1.89 μmol/g in the control group to 32.88 ± 2.67 μg/g in the lurasidone-treated group (p < 0.05), whereas the glutamine level remained relatively stable (13.13 ± 0.64 vs 12.95 ± 0.47 μg/g). This suggested that lurasidone specifically affected glutamate synthesis or metabolism rather than causing a generalized disturbance of the glutamate-glutamine cycle. The GABAergic system showed the most significant changes, with a dramatic decrease in GABA levels from 4.04 ± 0.71 to 2.83 ± 0.78 μg/g (p < 0.05). This significant reduction in a major inhibitory neurotransmitter is consistent with our previous observations of altered motor behavior and impaired tactile responses. At the same time, the maintenance of glycine levels suggested that the disruption of inhibitory neurotransmission was focused on the GABAergic pathway rather than a general effect on inhibitory signaling. In the monoamine system, dopamine and its metabolites showed synergistic changes. Dopamine concentrations decreased from 0.29 ± 0.01 to 0.25 ± 0.03 μg/g (p < 0.05), with corresponding decreases in its downstream metabolites 3-MT and metanephrine. These synergistic changes in the dopaminergic pathway corroborate with our transcriptomic findings, providing a mechanistic basis for the observed behavioral phenotype. The serotonergic system showed more complex alterations, with changes in both 5-HIAA and 5-HTP levels, but these changes did not reach statistical significance. These results suggested that the effects of Lurasidone on serotonergic signaling might be more subtle or compensated by regulatory mechanisms. The cholinergic and histaminergic systems showed slight perturbations, suggesting that the main effects of lurasidone were achieved through modulation of the amino acid and monoamine neurotransmitter systems, rather than extensive disruption of all neurotransmitter pathways. Importantly, while previous pharmacological characterizations of lurasidone have focused primarily on its receptor binding profile, our quantitative analysis reveals significant downstream metabolic consequences that have not been previously documented in developmental models. The notably severe GABA reduction (29.5%) compared to more moderate decreases in glutamate and dopamine (both 11%) suggests a preferential impact on inhibitory circuits that cannot be fully explained by lurasidone’s known receptor affinities alone. This disproportionate effect on GABAergic transmission represents a novel finding that may have implications for understanding both the therapeutic effects and potential developmental risks of lurasidone exposure.

## 4 Discussion

Lurasidone is a new atypical antipsychotic drug that acts mainly through high-affinity binding to dopamine D2 receptors and 5-hydroxytryptamine 5-HT2A receptors, and is used clinically for the treatment of schizophrenia and bipolar disorder (34). Lurasidone has lower metabolic side effects and is better tolerated than other antipsychotics (35, 36). However, its potential effects on the developing nervous system have not been fully investigated (37). This study investigated the potential neurodevelopmental toxicity of lurasidone using the zebrafish embryo model, revealing significant effects on early development, neurotransmitter systems, and behavioral patterns. Our results demonstrated that lurasidone exposure significantly affected zebrafish embryo development. The most notable findings included decreased survival rate at higher concentrations and developmental abnormalities, characterized by shorter body length and increased pericardial and yolk sac edema. These morphological changes were accompanied by significant behavioral alterations, as evidenced by decreased total swimming distance, average speed, and movement time. Of particular interest was the concentration-dependent reduction in both high mobile and normal mobile activities, suggesting compromised motor function. Noteworthy was the near-complete loss of touch response at higher concentrations, indicating potential disruption of sensory processing or motor output pathways. These behavioral changes correlate well with our neurotransmitter findings, particularly the significant reductions in glutamate, GABA, and dopamine levels.

The observed pattern of neurotransmitter alterations reveals several important contributions to the current understanding of lurasidone’s mechanism of action. While the reduction in dopamine levels (11%) is consistent with lurasidone’s known D2 receptor antagonism, the magnitude and pattern of changes in the glutamate-GABA system represent a previously uncharacterized aspect of its pharmacodynamic profile. The substantial decrease in GABA levels (29%) far exceeds the changes observed in other neurotransmitter systems and suggests that lurasidone’s effects on inhibitory neurotransmission are more profound than would be predicted based solely on its receptor binding profile. This disproportionate effect on GABAergic signaling may represent an indirect consequence of primary receptor interactions or could indicate previously unrecognized targets within the GABA synthesis or metabolism pathways. Our zebrafish model reveals for the first time the developmental consequences of these neurotransmitter alterations, linking molecular changes to specific behavioral deficits. The simultaneous disruption of both excitatory (glutamate) and inhibitory (GABA) signaling provides a novel explanation for the observed impairments in motor function and sensory integration. The relative preservation of serotonergic, cholinergic, and histaminergic systems despite significant changes in amino acid and catecholaminergic neurotransmitters suggests a more selective mechanism of action than previously appreciated. This selective pattern of neurotransmitter disturbance represents a unique neurochemical signature that distinguishes lurasidone from other atypical antipsychotics and may contribute to its clinical efficacy and side effect profile.

Our transcriptomic analysis revealed a complex pattern of gene expression changes, with particularly strong effects on circadian regulation and cell cycle-related pathways. The upregulation of circadian-related genes (including per1a) suggests that lurasidone may influence fundamental biological timing mechanisms, which could have broad implications for development and behavior. The downregulation of cell cycle-related genes, combined with changes in oxidative phosphorylation and ribosomal pathways, indicates that lurasidone may affect basic cellular processes necessary for normal development. The study demonstrated significant alterations in neurotransmitter levels, with reductions in glutamate, GABA, and dopamine. These changes provide a mechanistic link between transcriptomic alterations and behavioral deficits, suggesting that lurasidone affects neurotransmitter synthesis or release. The observed reductions in GABA and glutamate, key mediators of excitatory and inhibitory signaling, are particularly concerning as they imply disruptions in the delicate balance required for proper neural network formation and function. Dopaminergic changes further support the hypothesis of impaired sensorimotor integration and highlight the complexity of lurasidone’s impact on neurodevelopmental pathways.

Recent studies using rodent models, specifically rats subjected to chronic mild stress (CMS), have demonstrated that lurasidone, a multi-receptor modulator, significantly affects the expression of circadian rhythm-related genes such as Per1 and Bmal1 (38, 39). In these studies, CMS induced a depressive-like state accompanied by a reduction in BMAL1 and CLOCK protein levels in the prefrontal cortex, along with downregulation of several clock genes including Per1, Per2, Cry1, Cry2, and others, which is aligning with our observation of upregulated per1a and disrupted circadian pathways in zebrafish. Clinical studies have identified FKBP5 polymorphisms as predictors of early antipsychotic response (40, 41).

Our transcriptomic data revealed significant upregulation of fkbp5, a gene implicated in stress response and hypothalamic-pituitary-adrenal (HPA) axis regulation. While prior work in mammalian models has elucidated Lurasidone’s effects on neurotransmitter receptors (e.g., D2/5-HT2A), our zebrafish study provides the first multi-omics characterization of its developmental neurotoxicity. The integration of real-time behavioral tracking, transcriptomics, and neurotransmitter profiling offers a systems-level perspective.

While lurasidone is clinically valued for its efficacy in treating schizophrenia and bipolar depression, its potential developmental neurotoxicity warrants caution, particularly in vulnerable populations such as pregnant women and adolescents. The findings of this study align with broader concerns regarding psychotropic drug safety during critical developmental periods. The zebrafish model provides a robust platform for preclinical evaluation, but further studies are needed to validate these findings in mammalian models and to explore the underlying molecular mechanisms in greater detail.

## 5 Conclusion

In this study, we revealed the mechanism of developmental neurotoxicity of the antipsychotic lurasidone by integrating multidimensional analyses (morphology, behavior, transcriptomics and neurotransmitter assays) in a zebrafish embryo model. Experiments showed that lurasidone exposure at ≥4 mg/L significantly affected embryo survival and development: morphology showed shortened body length and abnormal pericardial/yolk sac dilatation; behavior detected reduced spontaneous movements and blunted tactile responses; and neurotransmitter systems showed simultaneous down-regulation of GABA (29%), dopamine (11%) and glutamate (11%). Transcriptome analysis revealed significant dysregulation of key genes of the circadian pathway and cell cycle regulatory genes. These findings suggest that lurasidone produces multiple damages to the nervous system during critical periods of development by interfering with neurotransmitter homeostasis in synergy with gene regulatory networks. The study highlights the need for a dynamic monitoring system for medication use during pregnancy and provides a multimodal research methodological framework for optimizing the developmental safety evaluation of psychotropic drugs.

## Data Availability

The data presented in the study are deposited in the NCBI GEO repository, accession number GSE299985.
