# Supplementary material for: Lurasidone induces developmental toxicity and behavioral impairments in zebrafish embryos
Source: Front Psychiatry. 2025 Jul 1;16:1581524. doi: 10.3389/fpsyt.2025.1581524 (PMC12259697; doi:10.3389/fpsyt.2025.1581524)
Supplement: Supplementary file 1 [file SupplementaryFile1.docx]

**Lurasidone Induces Developmental Toxicity and Behavioral Impairments in Zebrafish Embryos**

Wentian Li*^1^; Fang Wang*^3^; Zhe Feng*^4^; Qianqian Cheng^5^; Yuqing Huang^3^; Lisheng Zhu^4^; Han Xiao#^2^; Hongjian Gong#^2^

**Affiliations**

1. Department of Psychosomatic Medicine, Shanghai East Hospital, Tongji University School of Medicine,150 Jimo Road, Shanghai 200120, China, E-mail: liwentian2018@163.com

2. Institute of Maternal and Child Health, Wuhan Children’ s Hospital (Wuhan Maternal and Child Healthcare Hospital), Tongji Medical College, Huazhong University of Science & Technology, Wuhan 430016, Hubei, People's Republic of China, E-mail: [360683185@qq.com](mailto:360683185@qq.com)

3. Department of Psychosomatic Medicine, Wuhan Mental Health Center, Wuhan, 430022, Hubei, China.

4. School of Electrical Engineering and Automation, Hubei Normal University, Huangshi 435005, People's Republic of China, E-mail: zhefeng@hbnu.edu.cn

5. School of Mathematics and Physics, China University of Geosciences, Wuhan 430074, China.

* Contributed equally to this work

# Corresponding Author

Hongjian Gong, Institute of Maternal and Child Health, Wuhan Children’ s Hospital (Wuhan Maternal and Child Healthcare Hospital), Tongji Medical College, Huazhong University of Science & Technology, Wuhan 430016, Hubei, People's Republic of China. E-mail: [11407011@zju.edu.cn](mailto:11407011@zju.edu.cn)

Han Xiao, Institute of Maternal and Child Health, Wuhan Children’ s Hospital (Wuhan Maternal and Child Healthcare Hospital), Tongji Medical College, Huazhong University of Science & Technology, Wuhan 430016, Hubei, People's Republic of China

Tel: +86-0718-8263050, E-mail: [tjxiaohan1980@163.com](mailto:tjxiaohan1980@163.com)

Table S1. Primer sequences utilized for the RT-qPCR analysis.

| Gene symbol | Forward primer | Reverse primer |
| --- | --- | --- |
| Zf β-Actin | GATGCCCCTCGTGCTGTTTTC | TCTCTGTTGGCTTTGGGATTCA |
| Zf per1a | AACCGGTGCTTGTTTACCTG | TCTTCACCCTCAGGAGCAGT |
| Zf fkbp5 | ATCTGGCCCTGTGTTACCTG | TGCTTGAAGTCCATCAGTGC |
| Zf gngt2a | CGGGATATGTCCGATAAGGA | TCTGCAGATTGTGCCTCAAC |
| Zf ponzr5 | CTCGATGGATTTGGCTTCAT | GGAGCAAGCTCCACAGAAAG |
| Zf pnp5a | GGTGTGGAGACGGTGATTCT | AACCTTTCGTCGTTGTGTCC |


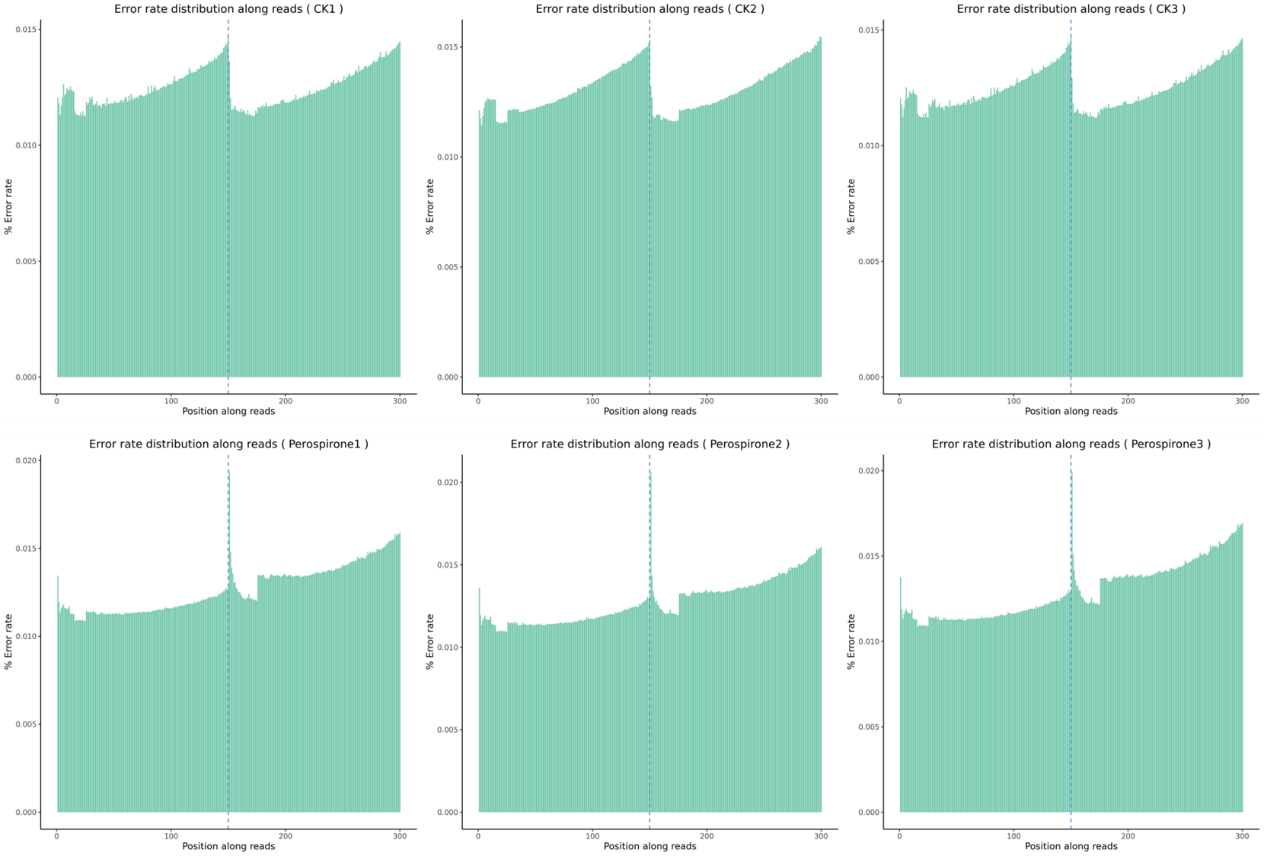


Fig. S1. Distribution of positional error rate for sequencing reads in both CK (control) and Lurasidone experimental groups.


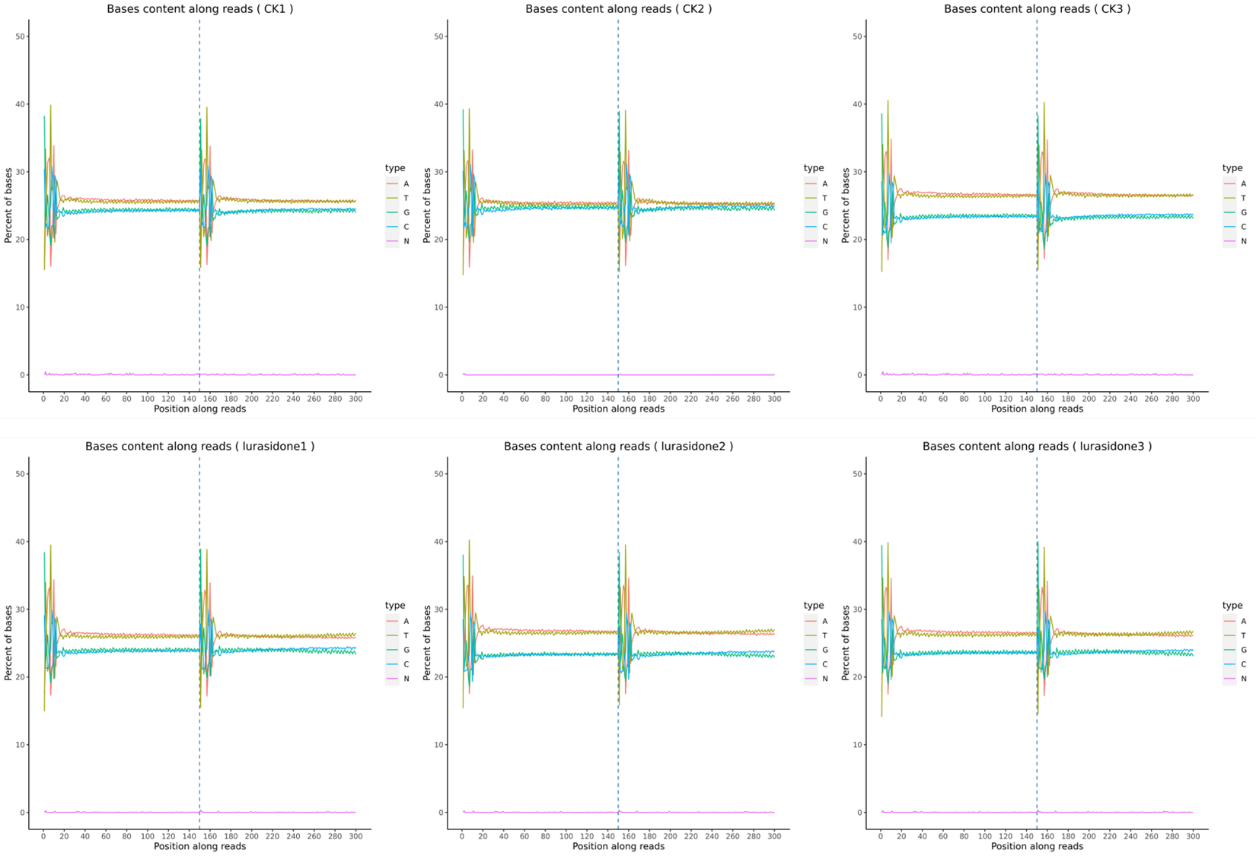


Fig. S2. Position-specific base composition analysis of sequencing reads in the CK (control) and Lurasidone groups.
